# Coordinated Evolution and Influencing Factors of Population and Economy in the Yangtze River Economic Belt

**DOI:** 10.3390/ijerph192114395

**Published:** 2022-11-03

**Authors:** Yazhu Wang, Hui Zou, Xuejun Duan, Lingqing Wang

**Affiliations:** 1 Key Laboratory of Watershed Geographic Sciences, Nanjing Institute of Geography and Limnology, Chinese Academy of Sciences, Nanjing 210008, China; 2 Institute of Geographical Sciences and Natural Resources Research, Chinese Academy of Sciences, Beijing 100101, China

**Keywords:** population, economy, coordinated evolution, influencing factors, Yangtze River Economic Belt

## Abstract

The degree of population–economy coupling and coordination is an important indicator of a region’s balanced development. This study examines the evolution of the population–economy coupling coordination pattern in the Yangtze River Economic Belt spanning from 2000 to 2019. It draws from the economic growth stage and related theories, and employs methods such as geographic concentration, center of gravity analysis, and the coupling coordination model. Accordingly, the population and economy of the Yangtze River Economic Belt form a core–periphery, with a decreasing center toward the periphery, and the east higher than the west. The spatial coupling situation of the population-economic center of gravity yields an inverted U-shaped curve, where their center of gravity separates and then converges, and the difference in regional development expands and then shrinks. Moreover, the population center of gravity lags behind that of the economy. The population–economy coupling and coordination degree shows a decreasing trend after rising fluctuations. Further, the study finds that regional economic development, government role, and market-led capital agglomeration are significant drivers of the population–economy coupling and coordination, with the industrial structural influence being spatially heterogeneous.

## 1. Introduction

Uncoordinated and unbalanced regional development is a prominent problem in China [1]. The excessive disparity has induced major negative effects on regional development. The regional discrepancy stems from the uncoordinated agglomeration of the economy and population in space.

There is a dialectical relationship between population and economic growth [2]. Population growth drives regional economic development by bringing a large number of labor and expanding the consumer market. At the same time, strong economic development momentum has become an important driving force for population growth. Regional economic development reacts on the population by increasing employment opportunities and improving living conditions. There is a positive causal relationship between population agglomeration and economic development. The spatial distribution and interaction of population and economy can explain the reasons for the imbalance of regional development. When the location, scale, and direction of population and productivity are basically the same, organizing production and living nearby can promote local economic development. Therefore, the spatial matching of population and economic factors has important practical significance for regional balanced development and optimization of spatial structure [3].

The population–economy interrelationship has long garnered much academic interest. Adam Smith notes that population growth is a sign of regional economic prosperity, whereas Malthus posits that excessive population growth inhibits economic development [4]. Marshall, Keynes, and Kuznets find a positive correlation between population and economy regarding agglomeration economy, demand and investment, and technological progress [5]. The theoretical methods of population and economic development mainly include neoclassical economics, push–pull model, quadratic index model, Lee’s migration law, etc. The main research methods include inconsistency index, geographical concentration, spatial autocorrelation, deviation index, and gravity center model [6,7,8]. The characteristics of the population and economic agglomeration and spatial interrelationships are analyzed at different spatial scales, such as national, urban cluster, inter-provincial, and urban [9,10]. Moreover, scholars show small scale, dynamic, diversification, and quantification trends [11]. The literature mainly examines the relationship between economic growth and scale and population structure, quality, aging, dividend, and spatial pattern [12,13], using theories such as Friedman’s central periphery, Williamson’s inverted U, Hirschman’s unbalanced growth, the spatial spillover effect, and economic gradient shift [14,15].

Relevant studies show that the higher the degree of coordination between population and economic coupling under resource allocation, the more balanced the regional development [1]. Given the combined influence of various factors such as historical foundations, factor flows, market mechanisms, and government policies, the spatial evolution of China’s population and economy has coupled and coordinated agglomeration dynamics [16,17]. Factors such as government regulation, market guidance, location, industrial structure, and technical level, influence the law of population, economic differentiation, and coupling coordination in different regions [18].

Hence, prior studies furnish an empirical foundation for the balanced and coordinated development of a population and economy, with relevant theoretical and methodological references [19,20]. However, they mostly examine China or a single province, studying areas with close internal connections and comparable development conditions. Therefore, research on multi-provincial regions with loose internal connections and significant differences in development levels is lacking [21]. On the other hand, the relevant research mainly focuses on the descriptive analysis of the pattern evolution of coupling coordination, while the quantitative research on the driving mechanism and influencing factors of coupling coordination is relatively lacking.

Accordingly, based on economic growth stage theories, this study employs geographic concentration, center of gravity analysis, and coupled coordination models to investigate the evolution of the coupling coordination pattern of population and economy in the Yangtze River Economic Belt from 2000 to 2019, and quantitatively analyze the factors that affect the population and economic coupling coordination. It is expected to formulate a reasonable population and economic development policy for the Yangtze River Economic Belt, which is cross provincial and has loose internal links and significant differences in development levels, and provide scientific reference for promoting the coordinated and balanced development and planning of the Yangtze River Economic Belt Basin.

## 2. Study Area and Methods

### 2.1. Study Area

**Figure 1:**
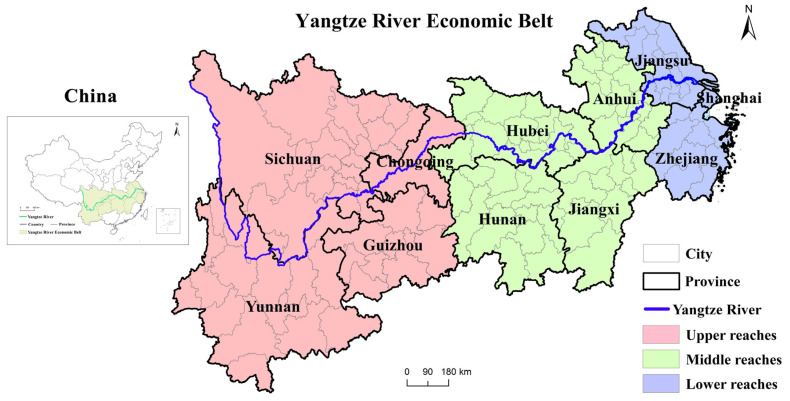
Survey map of the study area.

The Yangtze River is the largest river in Asia [22]. The Yangtze River Basin is a large economic belt with the largest population, largest industrial scale, and most complete urban system worldwide [23]. The research setting (Figure 1) includes 130 cities in nine provinces, and two municipal districts in the upper (Chongqing, Sichuan, Yunnan, and Guizhou), middle (Hubei, Hunan, Jiangxi, and Anhui), and lower (Shanghai, Zhejiang, and Jiangsu) reaches. It comprises three important national city groups (Chengdu-Chongqing, Yangtze River midstream, and Yangtze River Delta urban agglomerations) and regional urban agglomerations (e.g., the central Yunnan and Guizhou city groups). Its population and economy exceed 40% of the country’s total. The eastern, central, and western regions of the belt differ [24]. The eastern region accounts for 10.34% of the land area, with a high import and export trade volume of 80.28%. Therefore, the Yangtze River Economic Belt is adequate for regional development differences and coupling coordination [25].

### 2.2. Research Methods

#### 2.2.1. Geographical Concentration

Geographic concentration is an important index to measure the population–economy spatial pattern, considering the land area, population scale, and economic aggregate [26]. The population–economy geographical concentration degree measures the spatial distribution pattern and agglomeration situation of the population and economy in the belt as follows:

(1) $$R_{pop_{it}}=\frac{pop_{it}/POP_{t}}{land_{it}/LAND_{t}};R_{gdp_{it}}=\frac{gdp_{it}/GDP_{t}}{land_{it}/LAND_{t}}$$

where $R_{pop_{it}}$ is the population geographical concentration degree of city i in year t, $R_{gdp_{it}}$ is the economic geographical concentration degree of city i in year t, $LAND_{t}$ and $land_{it}$ are the total land area and land area of the city in year t, and $GDP_{t}$ and $gdp_{it}$ are the total GDP of the city and the GDP of the city in year t.

Furthermore, the coupling degree of geographical concentration can represent the coupling relation between population and economy [27].

(2) $$I=R_{{gdp}_{it}}/R_{pop_{it}}$$

where $R_{gdp_{it}}$ and $R_{pop_{it}}$ represent the degree of economic geographical concentration and population concentration, respectively. *I* represents the degree of coupling of geographical concentration: if *I* > 1, economic concentration exceeds population concentration, and it is the economically advanced type; if *I* = 1, it is the coordinated development of population and economic agglomeration, belonging to the coupled and coordinated type; if *I* < 1, population agglomeration exceeds economic agglomeration, and it belongs to the population advance type.

#### 2.2.2. Barycenter Analysis Model

Barycenter analysis is an important tool to study the spatial variation of regional elements by describing the spatial differences, dynamic changes, and evolutionary laws of geographical phenomena, such as barycenter points, barycenter movement direction, and movement distance [28]. The center of gravity of an attribute is usually calculated using an attribute and each administrative unit’s geographical coordinates. The barycenter model of population and economy is expressed as follows:

(3) $$X_{j}=\sum_{i=1}^{n}\frac{P_{ij}X_{i}}{\sum_{i=1}^{n}P_{ij}};\;Y_{j}=\sum_{i=1}^{n}\frac{P_{ij}Y_{i}}{\sum_{i=1}^{n}P_{ij}}$$

where ($X_{j}$,$Y_{j}$) is the population–economy coordinates in year j, $P_{ij}$ is the total urban population or economic aggregate in year j, and ($X_{i}$,$Y_{i}$) is the geographic center coordinates of the evaluation area. The migration distance model of population or economic center of gravity is

(4) $$D_{i-j}=R\times \sqrt{{\left(X_{i}-X_{j}\right)}^{2}+{\left(Y_{i}-Y_{j}\right)}^{2}}$$

where i and j represent two different years, $D_{i-j}$ represents the distance of the center of gravity moving between the two years, ($X_{i}$,$Y_{i}$) and ($X_{j}$,$Y_{j}$) are the spatial geographic coordinates of the demographic or economic center of gravity in the i and *j* years, and *R* is the constant term. The spatial movement direction model of population or center of gravity is

(5) $$\theta_{i-j}=\frac{n\pi }{2}+arctg\left(\frac{Y_{i}-Y_{j}}{X_{i}-X_{j}}\right)$$

where $\theta_{i-j}$ represents the moving angle of the center of gravity space in years i and j, with the direction due east being 0°, turning positive counterclockwise and negative clockwise. At 0° < θ < 90°, the center of gravity shifts northeast; 90° < θ < 180°, it shifts northwest; −90° < θ < 0°, southeast; and −180° < θ < −90°, southwest.

#### 2.2.3. Coupling Coordination Model

Drawing from the capacity coupling coefficient model in physics, He et al. [29] define the coupling function of population and economy as follows:

(6) $$C={\left\{\frac{\left(U_{p}\times U_{g}\right)}{\left(U_{p}\times U_{g}\right)\left(U_{p}+U_{g}\right)}\right\}}^{\frac{1}{2}}$$

where *C* is the coupling degree of population and economic development, and $U_{p}$ and $U_{g}$ are the level of population and economic development.

Population and economic development have an unbalanced dynamic nature. This study introduces the coupling coordination degree to reflect the real situation of the population and economic system [30]. The calculation is as follows:

(7) $$D={\left(K\times C\times T\right)}^{\theta };\;T=\delta U_{p}+\sigma U_{g}$$

where D is the degree of coupling coordination; K is the coordination coefficient and generally K=2; C is the degree of coupling; T is the comprehensive development index of economic development and population structure to characterize their benefits and levels; and θ*,* δ*,* σ are the development coefficients, generally considered 0.5, given the weight of equal importance of population and economy [31].

#### 2.2.4. Spatial Measurement Model

The ordinary least square (OLS) regression model is often used to quantitatively analyze the influence of independent variables on dependent variables [32]. Given spatial autocorrelation, the OLS model is biased. The spatial error model (SEM) and spatial lag model (SLM) of the maximum likelihood estimation method consider space factors that can optimize the regression model [33]. The SEM gauges the impact of the dependent variable error in the neighboring region on independent variables by discussing the spatial dependence of the disturbance error. The SLM discusses the spillover effect of each variable in the neighboring region [34].

(8) $$y=\rho W_{y}+X\beta +\varepsilon ,\varepsilon =\lambda W\varepsilon +\mu ,\varepsilon ∼\left(0,\sigma^{2}I_{n}\right)$$

where independent variable *X* is an n×k matrix, *y* is an n × 1 vector, ε is a random error term, λ and ρ are spatial autocorrelation coefficients, and *W* is an *n × n* spatial weight matrix.

This study uses human capital as a factor representing the market, and selected college students and the employed population to reflect the impact of human capital. Moreover, the government has introduced relevant policies to control the flow of resources and population to reduce the negative effects of regional differences. This study employs road area per square kilometer, per capita fiscal revenue, and fixed asset investment to reflect the government’s adjustable infrastructure and regional policy factors [35]. Regional economic development has geographical proximity and regional differences [36]. Location conditions are vital in population–economy coupling and coordination. The per capita GDP and foreign investment indicators characterize the impact of regional economic development on population and economic distribution [37]. Furthermore, industrial structure and transfer induce changes to the regional equilibrium pattern. The proportions of the secondary industry, tertiary industry, and urban–rural resident deposit balance represent the role of industrial structure factors [38].

### 2.3. Theoretical Analysis and Indicator System

**Figure 2:**
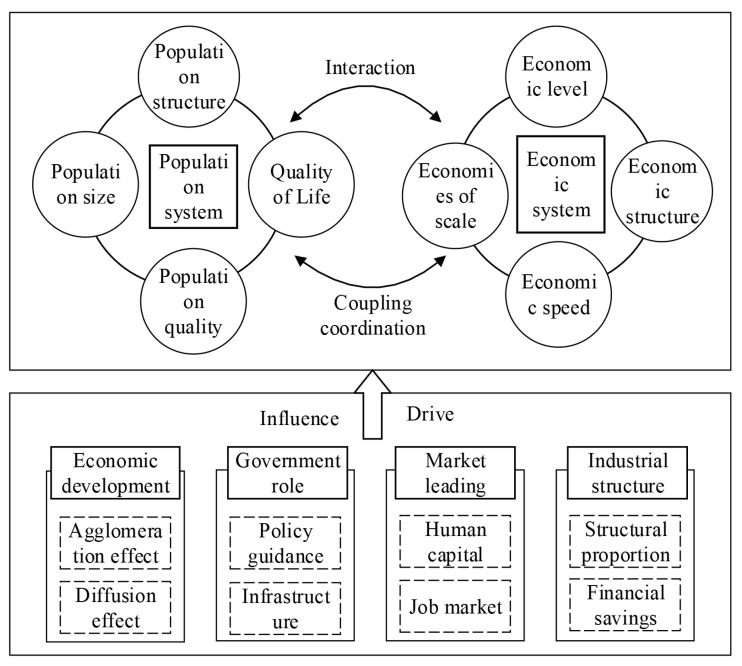
Driving mechanism of population and economy coupling coordination degree.

New economic geography believes that agglomeration economy makes industries highly concentrated in the net inflow area of population and capital, and promotes the self-strengthening “Matthew effect” of production factors such as population and capital, thus causing unbalanced regional development. Population and economy are affected by the role of market and government in the allocation of resources, thus affecting their coupling and coordination pattern. The market mechanism emphasizes high efficiency and guides the transfer of human resources and material capital to high profit areas. Through the “causal cycle” mechanism, industrial agglomeration is achieved. With the decrease of marginal benefits, the agglomeration effect turns into the diffusion effect, promoting the evolution of the balanced pattern of population and economic regions. At the same time, government regulation can also introduce relevant policies to actively intervene in the market, such as policy guidance and infrastructure construction, to reduce the loss of social and economic benefits caused by regional differences. In addition, based on the previous analysis, this paper uses population structure, population size, population quality, quality of life, economic level, economic scale, economic speed and economic structure to calculate the population economy coupling coordination. Referring to the existing relevant research, the theoretical framework of this paper is constructed (Figure 2).

In this paper, human capital is taken as a representative factor of the market. It selects the proportion of employed population and the proportion of college students to reflect the impact of human capital. In addition, the government has introduced relevant policies to regulate the flow of resources and population to reduce the negative effects of regional differences. This paper uses the road area per square kilometer, per capita financial income, and the ratio of fixed asset investment to reflect the infrastructure and regional policy factors that can be controlled by the government. Regional economic development has certain regional proximity and regional differences. Location conditions play an important role in the coupling and coordination of population and economy. GDP per capita and foreign investment indicators are used to characterize the impact of regional economic development on population and economic distribution. The industrial structure and industrial transfer will bring about changes in the regional equilibrium pattern. The proportion of the secondary industry, the proportion of the tertiary industry and the proportion of urban and rural residents’ savings deposits represent the role of industrial structure factors.

From the analyses of the basic connotation of the population and economic structure systems and the availability of data [39,40], the system of eight population indicators is constructed around population structure, size, quality, and life quality [41], from the aspects of economic level, scale, speed, and structure. Table 1 presents the evaluation index system of coupled and coordinated population–economy development. The study employs the extreme value method to normalize the data and eliminate the influence of the index dimension on the model.

### 2.4. Data Sources

This study employs data from the China City Statistical Yearbook (2001–2020) and the China Regional Economy Statistical Yearbook (2001–2020). The population system data employ the fifth (2000) and sixth (2010) national census data and 1% sample survey data. The population data of other years stem from the statistical yearbooks and bulletins of each city. Economic system data stem from statistical yearbooks and bulletins of various provinces and cities. For the missing data, the statistical bulletins of each city and county in the corresponding year are used as supplements, and the price index is used to offset all economic indicators to ensure the uniformity of indicators in different periods and regions.

## 3. Results and Discussion

### 3.1. Evolution of the Spatiotemporal Pattern

#### 3.1.1. Population Geographical Concentration

**Figure 3:**
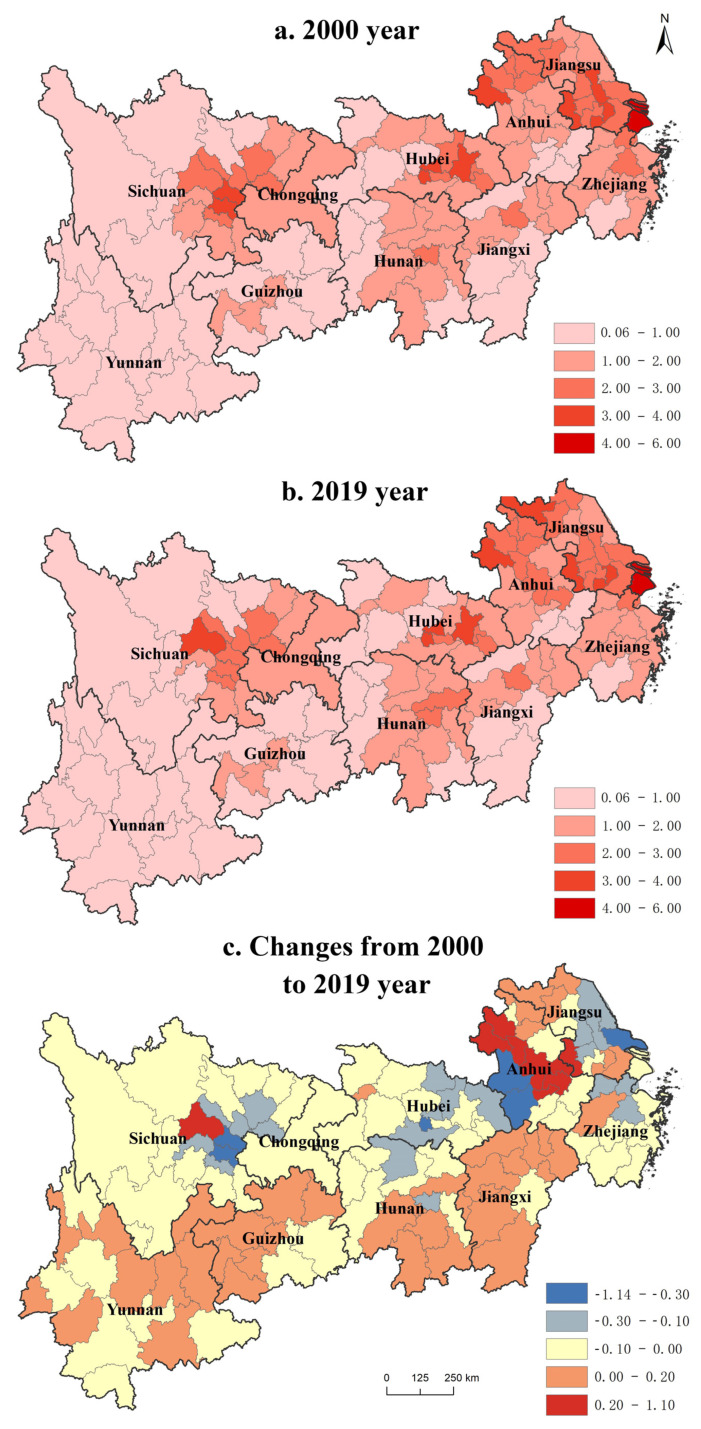
The evolution pattern of population geographic concentration from 2000 to 2019.

The population concentration in the Yangtze River Economic Belt (Figure 3) forms an obvious “core–periphery” structure, decreasing from the center to the periphery [42]. The uneven spatial pattern in the east is higher than that in the west. The urban agglomerations in the Yangtze River Delta, the middle reaches of the Yangtze River, and the Chengdu-Chongqing urban agglomeration are growth poles. The urbanization of most areas in Guizhou, Yunnan, and Sichuan is at a low-level development stage. The population agglomeration has a greater economic orientation. Furthermore, given natural conditions, historical foundations, national policies, regional differences, and constraints such as the household registration system and off-site employment, population changes are slow, and population distribution pattern changes are not significant, though the imbalance is increasingly significant.

From 2000 to 2019, with the influence of industrial transfer and policies, the population distribution pattern of the Yangtze River Economic Belt has undergone certain changes. Its population is expanding to central and western regions. The population of 38.46% of regions such as eastern Yunnan, western Guizhou, Jiangxi, and southern Hunan has continued to rise, and the polarization effect of provincial capital cities, such as Changsha, Nanchang, Guiyang, and Chengdu, is particularly prominent.

The urbanization demand of the central and western regions has induced significant population growth via the implementation of national strategies such as “Western Development” and the “Rise of Central China.” However, population agglomeration under policy control has strong regional selectivity. Cities with higher administrative levels receive more policy tilt via project approval and land indicators, which encourage population movement. Moreover, the major urban agglomerations in the Yangtze River Economic Belt show a circular layered distribution pattern. The population of the core cities of the Jianghuai and Chengdu-Chongqing urban agglomerations has expanded significantly, and the geographical concentration of the population has increased from 0.64 to 1.08. However, in a significant downward trend, the geographical concentration has dropped from 0.23 to 1.13, and the dual structure of urban and rural areas has been continuously strengthened. Given global industrial transfer, increasingly active markets, and social and economic development, the population flow tends to be multi-directional and active [43]. It is concentrated on the economically developed east coast, simultaneously shifting to some developed regions and provincial capitals in the west. The population movement in the central and western provinces is also active.

#### 3.1.2. Economic Geographic Concentration

**Figure 4:**
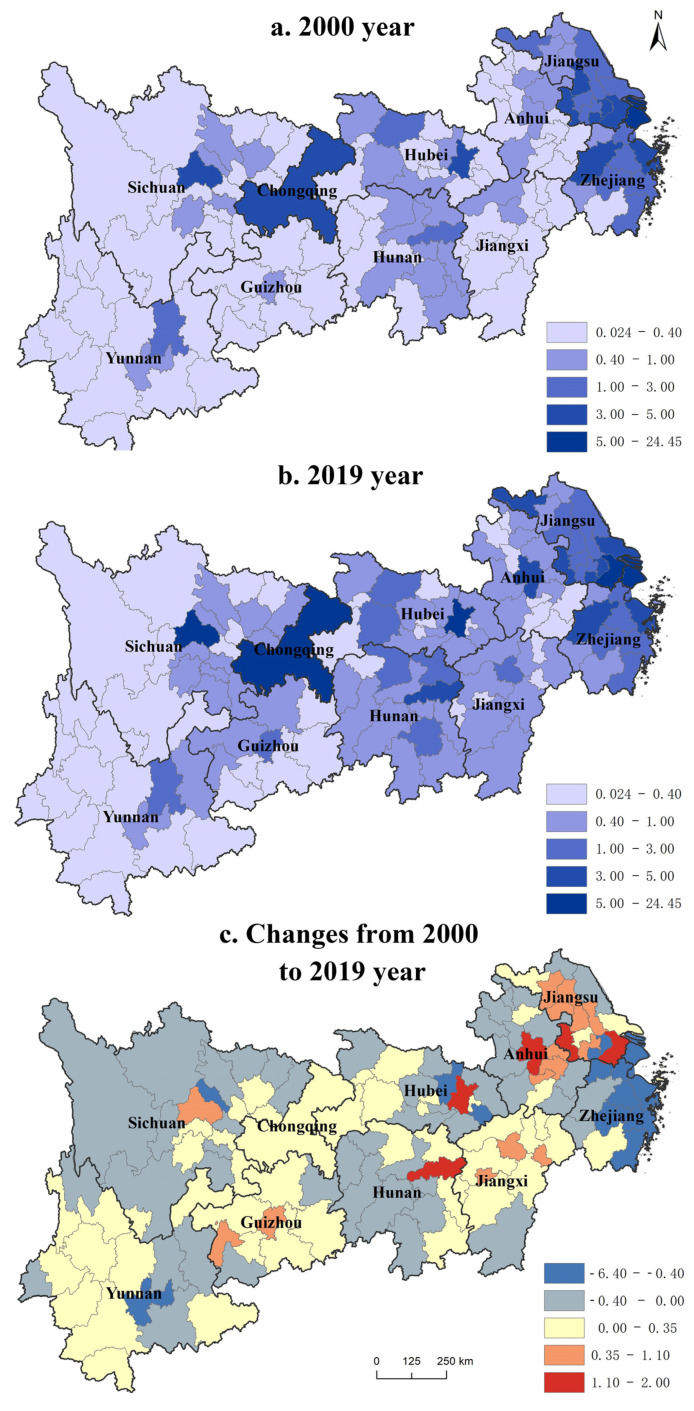
The evolution pattern of economic geographic concentration from 2000 to 2019.

The Yangtze River Economic Belt generally presents a stepped economic development spatial pattern with a developed east and backward west (Figure 4). The economic geographic concentration of the Yangtze River Economic Belt gradually decreases from the lower reaches to the upper reaches. The Yunnan Guizhou and Western Sichuan economy in the upper reaches of the Yangtze River is relatively backward, according to Li et al. [44]. The pattern of economic concentration shows a striped distribution feature decreasing from east to west, with significant differences between the upper, middle, and lower reaches. The three growth poles in the Yangtze River Economic Belt are significant in the Yangtze River Delta, Wuhan, and Chengdu-Chongqing urban agglomerations, with spatial spillover effects [45].

From a temporal evolutionary perspective, the geographic concentration of the Yangtze River Economic Belt shows a rising first and then falling trend, rising from 1.65 in 2000 to 1.69 in 2010, and then falling to 1.66 in 2016. Such economic development shows a “balanced–unbalanced–gradually-balanced” trend, as per Williamson’s “inverted U-shaped” regional economic development theory. From the spatial evolution perspective, from 2000 to 2015, the primary manifestation is that the geographical concentration of provincial capital cities (e.g., Nanjing, Hefei, Wuhan, Guiyang, and Chengdu) has increased significantly, ranging from 0.47 to 1.98. Such provincial capitals continue to attract production factors such as capital and technology from surrounding cities, and the agglomeration effect is prominent. However, the economic concentration of Shanghai and southern Zhejiang has dropped significantly, and the drop in Shanghai is as high as 6.44. As an international economic center, Shanghai has not fully promoted the Yangtze River Economic Belt. Western regions such as Western Sichuan, Western Yunnan, and Southern Guizhou have also declined. The national “Rise of Central China” and “Western Development” strategies have begun to take effect [46]. Economic growth has expanded from the eastern coastal areas to inland areas along the Yangtze River, and the regional economic gap in the Yangtze River Economic Belt has decreased, promoting a balanced economic development of the basin [47].

### 3.2. Spatiotemporal Evolution of Coupling Coordination Degree between Population and Economy

#### 3.2.1. Population and Economic Development Stage Based on the Barycenter Model

**Figure 5:**
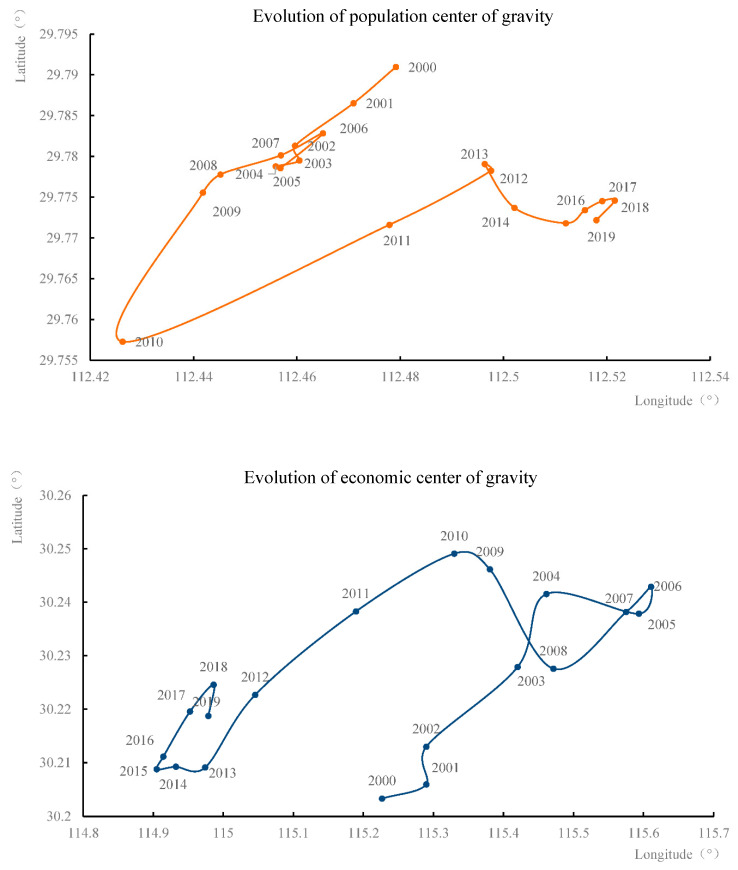
Evolution of population and economic center of gravity from 2000 to 2019.

The spatial evolution and coupling situation of the population and economic center of gravity were measured based on the attributes and geographic data of the spatial unit of the Yangtze River Economic Belt from 2000 to 2019 (Figure 5). The reasons for the change and its relationship with the balanced development of regional space were analyzed. The spatial coupling situation of the population and economic center of gravity conforms to the “inverted U-shaped” curve; that is, the population and economic center of gravity present a process of separation and then convergence, and the difference in regional development shows a trend of first expanding and then shrinking. The moving range of the economic center of gravity (68.04 km) is greater than that of the population center of gravity (9.10 km), with the “Hu Huanyong Line” likely having spatial stability.

Furthermore, population migration is subject to multiple resistances such as the restrictions of the household registration system, family planning policies, and challenges in employment, inducing relatively slow changes in its magnitude and a mutual incongruity between population and economic center of gravity shifts. The deviation distance between the center of gravity of the population and economy shows a decreasing trend; for example, in 2000, it was 269.52 km, decreasing to 237.21 km in 2016. The population–economy fluctuation tends to ease, and the regional spatial structure of the Yangtze River Economic Belt becomes stable. The large-scale migration of the population and economic center of gravity, and the regional development implemented in various stages of the Yangtze River Economic Belt are in a certain degree of spatial consistency, with a slight time lag. Policy factors are an important external force that promotes population agglomeration and economic development. Further detailed analysis of the evolution pattern of the population and economy in different stages from 2000 to 2019 and the coupling of regional economic development policies can be divided into three development stages.

In the first stage (2000–2005), the population and economic center of gravity of the Yangtze River Economic Belt were separated. The center of gravity distance between the two increased from 269.25 to 307.84 km. The population center of gravity shifted to the southwest by 1.64 km, while the economic center of gravity shifted to the northeast by 37.58 km. The central government proposed the “Yangtze River Development Strategy”, leading the Yangtze River Delta development with the development of Shanghai. The provinces along the Yangtze River generated a strong sense of development, shifted the focus of urban development to the Yangtze River, and introduced investment policies to accord with the Shanghai development policy, resulting in the rapid development of the export-oriented economy, as reflected in the economic center of gravity moving to the northeast. China’s household registration policy restricts the population flow from rural areas (small towns) to cities (high-end cities), hindering the optimization of population space. Many laborers cannot settle down and enjoy the same social security benefits as local residents. They can only travel to and from their workplace and hometown in a tidal manner. The population and economic centers of gravity show a tendency to separate, the gap in regional development widens, and the spatial distribution is uneven.

In the second stage (2006–2009), the population center of gravity continued to shift to the southwest by 4.70 km, while the economic center of gravity shifted to the southwest by 27.01 km. The population and economic center of gravity shifted in the same direction along a close trend, and the distance between the two reduced to 286.05 km. The country implemented development strategies such as the “Rise of Central China” and “Western Development” to narrow the growing regional differences for a regional and balanced economic development [48]. Beyond the existing Yangtze River Delta urban agglomeration, the Yangtze River also formed Chengdu-Chongqing and Poyang Lake Ecological Economic Zones, a Wuhan urban agglomeration, and other polarized areas and urban continuous belts [49]. The river basin economy evolves from point-like to urban agglomeration development. The economy radiates from the lower to the middle and upper reaches from the east to the west. The population and economic center of gravity shifted to the southwest, narrowing the regional development difference.

In the third stage (2010–2019), the economic center of gravity continued to shift westward by 40.18 km, while the population center shifted eastward by 8.85 km. Both centers of gravity moved ever closer, further reducing the distance to 236.84 km. The urbanization of the lower reaches of the Yangtze River proceeds rapidly, and the population center of gravity shifts to the east, presenting the “Matthew Effect” spatial polarization mode. The Yangtze River Economic Belt is formally a national strategy and among the new-era’s “three supporting belts” determining the development strategy of “all efforts to protect and not develop.” Given China’s focus on the balanced economic development of the Yangtze River Basin, the belt has entered a period of economic structural transformation. Moreover, with the industrial structure optimized, the regional imbalance weakens, and the economic order–scale structure becomes balanced.

#### 3.2.2. Spatiotemporal Evolution of Coupling Coordination Degree

**Figure 6:**
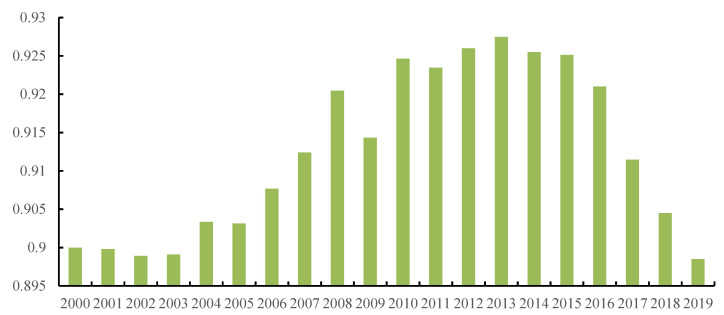
Degree of coordination between population and economy from 2000 to 2019.

The coupling coordination degree of population and economy in the Yangtze River Economic Zone generally shows a rising trend after fluctuations (Figure 6) from 0.890 in 2000 to 0.927 in 2013, and a drop to 0.910 in 2019. It shows that the distribution of population and economy in the Yangtze River Economic Belt gradually balances over time and then becomes unbalanced. From 2000 to 2003, the coupling and coordination of population and economy slowly declined because agriculture dominated most of the Yangtze River Economic Belt at the beginning of the 21st century, with low economic development, low urbanization, and excessive population, which restricted economic development. The population–economy contradiction is sharp, and the degree of coupling and coordination declined. The global economic situation was good from 2003 to 2008, and the Yangtze River Economic Belt ushered in a golden period of rapid growth. Under the guidance of regional background development conditions, the economic growth of large cities and provincial capitals was faster than that of small cities, and labor-intensive enterprises attract population agglomeration. Living standards, lifestyles, and education and fertility concepts have, meanwhile, changed with economic development, population numbers were under control, and the population–economy contradiction eased. The industrial structure adjustments, urbanization transformation, and educational reform continuously promote the coordinated development of the population economy, and the degree of coupling and coordination show a rapid growth trend. In 2009, given the changes in the external environment and the financial crisis, production cost increased, and the degree of coupling and coordination declined concavely [50]. After 2013, the economic growth slowed down. Given regional development environment and industrial transfer changes, the economic development did not attract rapid population agglomeration and transfer in underdeveloped areas. The population and economy gradually became unbalanced, with regional differences remaining. Improvement in the economic development stage promotes the coupling and coordination upgrading of the population and economic system. However, the complex relationship between population and economic system may make the coupling and coordination degree repetitive.

**Figure 7:**
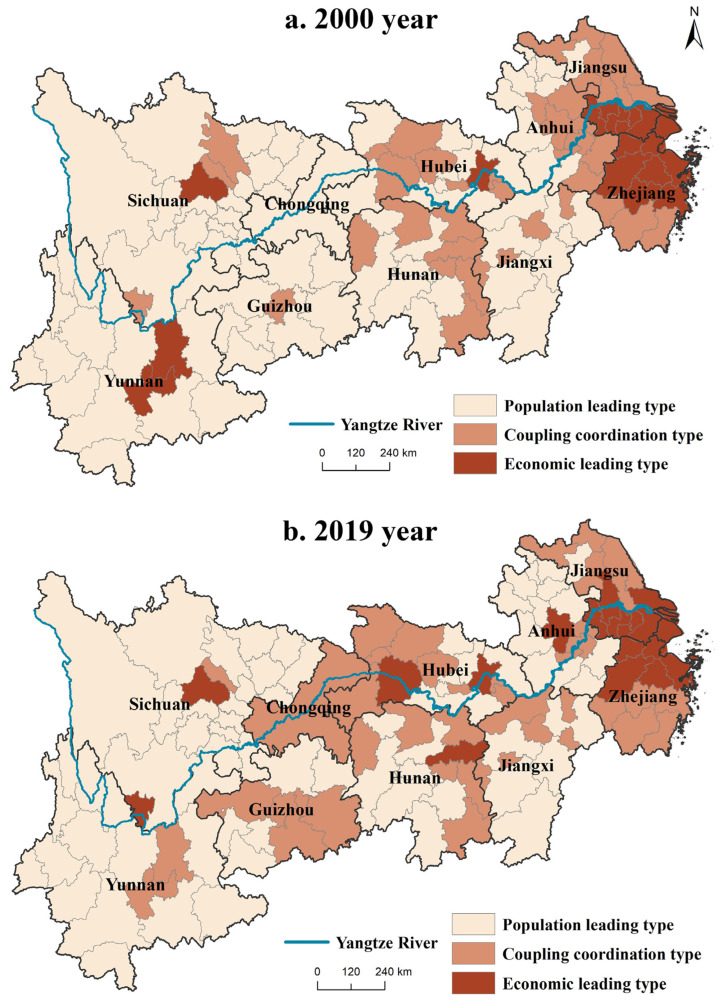
The degree of coordination between population and economy in 2000 and 2019.

There remain significant spatial differences in the coupling coordination degree pattern of the Yangtze River Economic Belt, which can be divided into three types: population-leading, coupling coordination, and economic-leading (Figure 7). From 2000 to 2019, the population and economic coupling coordination types were mainly economic- and population-leading types, accounting for 33.84% and 58.46% of the total, respectively. The coupling coordination type accounted for 7.7%. The economic-leading type is mainly distributed in the Yangtze River Delta, Chengdu-Chongqing, and Chang-Zhu-Tan urban agglomerations. Given urbanization and industrialization, the region has seen rapid socio-economic development while attracting large populations. However, given the impact of the household registration system, population transfer is not complete, population attraction has a certain lag, and the economic concentration rate is faster than the population concentration. Population-leading cities are mainly distributed in provinces such as Yunnan, Guizhou, and Sichuan with poor economic foundations and backward economic development but with a high population growth rate. Furthermore, the population employment structure lags the industrial structure. Alternatively, the population quality is not high enough to meet the needs of economic development. From the perspective of spatiotemporal evolution, during the 2000–2019 period, the main structure of the coupling and coordination of the population and economy has not changed, but the spatial distribution and quantity of various types have changed, mainly from the original coupling and coordination type reduction to economic leading type, accounting for 4.62% of all types.

### 3.3. Analysis Influencing Factors of Population and Economy Coupling Coordination

#### 3.3.1. Model Setting and Testing

Moran’s I (error) of the Yangtze River Economic Belt is 4.1601; thus, the coupling coordination degree between the population and economy has spatial autocorrelation (Table 2). The middle and lower reaches have spatial autocorrelation, while the upper reaches have no obvious spatial effect. The rook-based LM (lag) of the spatial weight matrix is 19.5693, greater than 11.8401 of LM (error). LM (lag) is more significant than LM (error), both passing the 1% significance level. Thus, the SLM is more desirable for the belt. The SEM in the middle reaches is more desirable. For the lower reaches, the queen-based LM (lag) of the spatial weight matrix is 17.0605, greater than the LM (error) value of 3.1550. LM (lag) and RLM (lag) pass the 1% significance level; hence, the SLM for the lower reaches is preferable. For the middle reaches, the SEM (error) passed the significance test at the 10% level of the spatial weight matrix based on rook, while the SLM failed the significance test. Therefore, the SEM for the middle reaches is more suitable. However, for the upper reaches, the econometric models fail to pass the test.

#### 3.3.2. Model Regression Results

The test results showed that (Table 3), by comparing OLS with SLM and SEM models, all test values of SLM and SEM improved relative to the OLS model without considering spatial autocorrelation. Thus, the economic model controlling spatial lag and dependence is more optimized. Regarding the test value of the Yangtze River Economic Belt, for example, the goodness of fit R2 increases from 0.8251 to 0.8532, logL increases from −26.5947 to −16.4784, and AIC and SC decrease from 75.1894 and 106.7321 to 56.9568 and 91.3672, respectively, proving that SLM and SEM are preferable, given that the spatial effect improved the fitting degree of the model. However, the OLS corresponding test values have a relatively poor effect and are not desirable. The spatial hysteric coefficient ρ and the spatial error coefficient λ are significantly positive at the 1% level. Thus, the spatial effect of the coupling coordination degree between the population and economy in the Yangtze Economic Belt is reflected by the spillover effect of neighboring cities, and the error impact also has a certain effect on the coupling coordination degree.

From the regression results, the characteristics of the spatial differences of each element induce the spatial pattern evolution and spatial imbalance of the coupled and coordinated development of the population and economic system. Per capita GDP, employed population, fixed asset investment, highway density, per capita fiscal income, and the proportion of urban–rural resident deposit balance have significant positive influences on the coupling coordination degree. However, the secondary production, tertiary industry, college student, and foreign investment proportion strengthen the population–economy contradiction. Regional economic development, market forces, industrial structure, and government policies significantly impact the coordination degree of coupling between population and economy [37].

Regional economic development is a fundamental driver [51]. It promotes the population–economy coupling coordination with the greatest sensitivity, the most being the influence of the lower reaches of the Yangtze River. The coupling coordination degree increases by 0.54% for every percentage point increase in per capita GDP of the Yangtze River Economic Belt, while that of the lower region increases by 0.69%. The lower reaches have obvious geographical advantages, undertake the industrial transfer of the global production network, and attract various resource factor agglomeration such as capital and technology, thus promoting the high-level agglomeration of the population and economy, as per Mohsen and Chua [52]. Population spatial agglomeration is the result of economic agglomeration and regional economic differences, which induce migration into economically developed areas for better living conditions, further concentrating the population therein for regional coordinated development. Several spatial polarizations trickle-down, and cyclic cumulative causal effects affect the coupling coordination pattern. The regional economic development level of the Belt is the fundamental driver of the coordination degree of population–economy coupling.

The role of government is also an important driving force. The government acts on the agglomeration and population and economic development through fiscal revenue and fixed asset investment. The influence coefficients of per capita fiscal income and the proportion of fixed asset investment are 0.1859 and 0.0256, while those in the lower reaches of the Yangtze River are as high as 0.9628 and 0.2222. Financial means and fixed asset investment can promote urban construction and the steady improvement of urban competitiveness. They can also reduce regional financial and asset differences to promote regional balance and improve social welfare to attract population inflow and promote the coordinated population–economy development. Moreover, the infrastructure built by government departments, such as road density, has a certain impact. Cities with convenient transportation are more likely to accept industrial transfer; that is, traffic accessibility has a significant positive impact on economic growth and population agglomeration, according to Jacobs-Crisioni and Koomen [53].

Market-led capital accumulation is another important driver [54]. The proportion of the employed population is significantly and positively correlated with the degree of coupling coordination, especially in the middle and lower reaches of the Yangtze River, where the correlation coefficients are as high as 1.58 and 1.32. Given that the Yangtze River Economic Belt is dominated by labor-intensive and capital-intensive manufacturing industries, it is highly attractive to the employed population, and human capital strengthens the coupling and coordination degree between population and economy. Moreover, whether the employment growth can be promoted is vital to whether the city promotes population agglomeration. The classical and new economic growth theories regard the capital accumulation led by the market as an important driver of population agglomeration and economic growth [55].

The influence of industrial structure is spatially differentiated [56]. Residents’ increased balance can promote a sense of security, allow enterprise production and business operation via bank loans, and reduce consumption via high household savings, destroying the balance between savings and consumption. Given the reduced social-economic activities and rising unemployment, inducing an uncoordinated population and economy, residents’ balance and coordination degree have a high negative correlation of 0.1632. The structural ratio of secondary and tertiary industries has a low correlation with the degree of coupling coordination. The middle reaches and the Yangtze River Economic Belt have a negative correlation, but the lower reaches show a positive correlation. There, a strong economic agglomeration capacity induces economic development. The industrial structure adjustment of the secondary and tertiary industry proportion can promote a rapid increase in the urban population and economy [57], and improve the coupling and coordination degree. The scale economy effect by industrial agglomeration is an important driver of regional, coordinated, and balanced development.

## 4. Conclusions

A large number of documents have studied the relationship between population and economy. However, the research on the driving mechanism of spatial coupling and coordination between population and economy is still insufficient. In order to fill this research gap, we focus on the spatiotemporal response of population and economic coupling coordination and its influencing factors. The main conclusions are as follows:

There is a spatial difference in the pattern of population and economic geographic concentration in the Yangtze River Economic Belt. The geographic concentration of population presents a “core–periphery” unbalanced spatial pattern of “high in the east and low in the west,” while the degree of economic geographic concentration highlights a more obvious “center–periphery” pattern. Regarding spatiotemporal evolution, affected by national strategies such as “Western Development” and “Rise of Central China,” the population shows an expanding trend to the central and western regions. The economic development presents a “balanced–unbalanced–gradually balanced” situation.

The spatial coupling of the population and economic center of gravity conforms to the “inverted U-shaped” curve. The population and economic center of gravity present a process of separation and then convergence. The difference in regional development shows a trend of first expanding and then shrinking. The population center of gravity first shifts to the southwest and then to the northeast. The economic center of gravity shifts first to the northeast and then to the southwest. Population migration has spatial lag and stability.

The degree of coupling and coordination between the population and economy shows a falling after rising fluctuation trend. The higher the economic development stage, the higher the degree of coupling, and the coupling coordination index and economic development level remain spatially consistent. Factor differences affect population–economy coupling and coordination in different regions. Regional economic development, market forces, industrial structure, and government policies significantly impact such coupling and coordination, where the influence of industrial structure is spatially heterogeneous.

The coupling and coordinated development of population and economic system is the result of complex interaction of many factors. Future studies can probe the interaction mechanism of population mobility and economic development, and examine the complex relationship between population and economy and the drivers and countermeasures of coupling coordination.

## Figures and Tables

**Table 1:** Comprehensive evaluation index system for coupled and coordinative population–economy development.

First-Level Index	Second-Level Index	Third-Level Index	Unit
Population system	Population size	Population	Ten thousand people
		Natural growth rate	%
	Population structure	Proportion of non-agricultural employment	%
		Proportion of working population	%
	Population quality	Number of students in general higher education	Ten thousand people
		Number of college students per 10,000 people	People
	Quality of Life	Urban per capita disposable income	CNY
		Per capita net income of rural residents	CNY
Economic system	Economies of scale	GDP	100 million CNY
		Total investment in fixed assets	100 million CNY
	Economic level	Per capita GDP	CNY
		Total retail sales of consumer goods	100 million CNY
	Economic structure	Output value of the secondary and tertiary industries in GDP	100 million CNY
		Local fiscal revenue in GDP	%
	Economic speed	GDP growth rate	%
		Growth rate of local revenue	%

Note. CNY = Chinese Yuan; GDP = gross domestic product.

**Table 2:** Spatial dependence test.

Test	Yangtze River Economic Belt		Upper Reaches		Middle Reaches		Lower Reaches	
	Value	Prob	Value	Prob	Value	Prob	Value	Prob
Moran’s I (error)	4.1601	0.0000	−0.0598	0.9523	2.7561	0.0058	2.4147	0.0157
LM (lag)	19.5693	0.0000	0.7873	0.3749	1.0331	0.3094	17.0605	0.0000
RLM (lag)	9.3518	0.0022	0.5017	0.4787	0.0019	0.9656	14.2621	0.0002
LM (error)	11.8401	0.0006	0.3005	0.5835	4.1664	0.0412	3.1550	0.0757
RLM (error)	1.6226	0.2027	0.0150	0.9026	3.1352	0.0766	0.3567	0.5503

**Table 3:** OLS, SLM, and SEM of the spatial measurement model results.

	Yangtze River Economic Belt		Middle Reaches		Lower Reaches	
	OLS	SLM	OLS	SEM	OLS	SLM
CONSTANT	−10.0096 ***	−8.5503 ***	−7.4373 ***	−7.1038 ***	−15.0356 ***	−13.9955 ***
Per capita GDP	0.6250 ***	0.5454 ***	0.5067 ***	0.4539 ***	0.4962	0.6943 ***
Employed population	0.3514 **	0.3163 ***	1.8502 ***	1.5758 ***	0.7912	1.3246 **
Secondary industry	−0.0063	−0.0078	−0.0103	−0.0071	0.0445	0.0317 **
Tertiary industry	0.0014	−0.0038	−0.0022	−0.0039	0.0388	0.0379 **
Fixed assets investment	0.0016	0.0256	0.3152 ***	0.2254 **	0.0080	0.2222 **
Road area	0.0369 ***	0.0334 ***	0.0218	0.0242 *	0.0432 **	0.0698 ***
College students	−0.0010	0.0020	0.0035 *	0.0040 ***	−0.0047	−0.0121 ***
Fiscal revenue	0.2115 ***	0.1859 ***	0.0612	0.1034	0.8907 ***	0.9628 ***
Residents deposit balance	0.2003 **	−0.1632 **	−0.0963	−0.0136	−0.0259	−0.1123
Foreign investment	−0.0453 *	−0.0619 ***	−0.0788 ***	−0.0791 ***	−0.0328	0.0599 *
R2	0.8251	0.8532	0.8904	0.9053	0.9738	0.9899
logL	−26.5947	−16.4784	28.6565	31.4108	12.1891	23.8009
AIC	75.1894	56.9568	−35.3131	−40.8217	−2.37813	−23.6018
SC	106.7321	91.3672	−12.6482	−18.1568	11.0295	−8.9753

Note. OLS = ordinary least square; SLM = spatial lag model; SEM = spatial error model; GDP = gross domestic product; AIC = Akaike information criterion; SC = Schwarz criterion. ***, **, and * represent significance levels of 1%, 5%, and 10%, respectively.

## Data Availability

The data used to support the findings of this study are available from the corresponding author upon request.
